# CRISPR/Cas9-mediated lipoxygenase gene-editing in yellow pea leads to major changes in fatty acid and flavor profiles

**DOI:** 10.3389/fpls.2023.1246905

**Published:** 2023-09-21

**Authors:** Pankaj Bhowmik, Wei Yan, Connor Hodgins, Brittany Polley, Tom Warkentin, Michael Nickerson, Dae-Kyun Ro, Frédéric Marsolais, Claire Domoney, Shiva Shariati-Ievari, Michel Aliani

**Affiliations:** ^1^Aquatic and Crop Resource Development Centre, National Research Council Canada, Saskatoon, SK, Canada; ^2^Department of Biological Sciences, University of Calgary, Calgary, AB, Canada; ^3^Department of Plant Sciences, University of Saskatchewan, Saskatoon, SK, Canada; ^4^Department of Food and Bioproduct Sciences, University of Saskatchewan, Saskatoon, SK, Canada; ^5^London Research and Development Centre, Agriculture and Agri-Food Canada, London, ON, Canada; ^6^Department of Biochemistry and Metabolism, John Innes Centre, Norwich Research Park, Norwich, United Kingdom; ^7^Division of Neurodegenerative Diseases (DND), St Boniface Hospital Research Center, Winnipeg, MB, Canada; ^8^Department of Food and Human Nutritional Sciences, University of Manitoba, Winnipeg, MB, Canada

**Keywords:** pea, gene-editing, CRISPR/Cas9, lipoxygenase, fatty acids, aroma, flavor

## Abstract

**Introduction:**

Although pulses are nutritious foods containing high amounts of protein, fiber and phytochemicals, their consumption and use in the food industry have been limited due to the formation of unappealing flavors/aromas described as beany, green, and grassy. Lipoxygenase (LOX) enzymes are prevalent among pulse seeds, and their activity can lead to the formation of specific volatile organic compounds (VOCs) from certain polyunsaturated fatty acids (PUFAs). As a widespread issue in legumes, including soybean, these VOCs have been linked to certain unappealing taste perception of foods containing processed pulse seeds.

**Methods:**

To address this problem in pea and as proof of principle to promote the wider use of pulses, a Clustered Regularly Interspaced Short Palindromic Repeats (CRISPR) construct was designed to create null alleles (knockouts) of *PsLOX2* which had been implicated in the generation of VOCs in peas.

**Results and discussion:**

Successful CRISPR/Cas9-mediated LOX gene editing of stable transgenic pea lines (TGP) was confirmed by DNA sequencing of the wild type (WT) and TGP *pslox2* mutant lines. These lines were also assessed for LOX activity, PUFA levels, and VOCs. Compared to WT peas, the TGP lines showed a significant reduction (p < 0.05) in LOX activity and in the concentration of key VOCs, including hexanal, 2-hexenal, heptanal, (E)-2-heptenal, (E,E)-2,4-heptadienal, 1-octen-3-ol, octanal, (E)-2-octenal (E,E)-2,4-nonadienal and furan-2-pentyl. The content of two essential PUFAs, linoleic and α-linolenic acids, the known substrates of LOX in plants, was higher in TGP flours, indicating the efficacy of the CRISPR-mediated gene editing in minimizing their oxidation and the further modification of PUFAs and their products. The collection of VOCs from the headspace of ground pea seeds, using a portable eNose also distinguished the TGP and WT lines. Multiple regression analysis showed that LOX activity correlated with the two VOCs, heptanal and (E,E)-2,4-heptadienal in pea flours. Partial Least Squares Regression (PLS-R) plot for selected PUFAs, VOCs, and sensor responses in WT and TGP lines showed distinct clusters for WT and TGP lines. Together this data demonstrates the utility of CRISPR mediated mutagenesis of *PsLOX2* to quickly improve aroma and fatty acid (FA) profiles of pea seeds of an elite Canadian variety.

## 1 Introduction

Canadian pea production for 2020 was 4.6 million tonnes accounting for nearly one-third (31%) of the total global production, followed by Russia and China (FAOSTAT, 2020). Yellow pea is one of the key pulses grown in Western Canada and elsewhere with considerable interest from the food industry. Although an excellent source of protein, in common with other legumes, yellow pea products face sensory challenges relating to their acceptability and stability over time. A major contributor to these challenges is a group of volatile organic compounds (VOCs) generated from both enzymatic and non-enzymatic chemical reactions during thermal processing which give pulse products unpleasant aromas (e.g., beany, green, grassy) and off-flavors (Ma et al., 2015). The enzymes that generate VOCs in peas and other pulses are lipoxygenases (LOX) – and alcohol oxidoreductases – that convert the polyunsaturated fatty acids (PUFAs), linoleic and α-linolenic acids, to aldehydes and a range of alcohols (Jelen and Wasowicz, 2011).

In recent years, it has been demonstrated that even partial deactivation of LOX activity using heat treatments such as micronization (Shariati-Ievari et al., 2016) and RevTech (RT), a patented heat treatment and pasteurization process (Fahmi et al., 2019), was successful in minimizing the generation of the key VOCs responsible for off-aroma and flavors of cooked legume flours (lentils, chickpeas, red beans, and yellow peas). A significant decrease in LOX activity in split yellow pea flours has also been reported when treated with RevTech at 140°C with 10% steam (RT10%) and bread samples fortified with the treated split yellow pea flour were more acceptable when compared to bread with untreated pea flour or pea flour with RT at 140°C without steam (RT0%) (Fahmi et al., 2019). These results suggested that the total or partial inactivation of LOX enzymes in RT10% pea flours was key to the consumer acceptability results observed. The data generated suggest that the thermostability of the enzyme varied significantly depending on the product type, heat treatment condition, and evaluation assays used.

Although processing studies have indicated the effectiveness of heat in reducing LOX activity in legumes (Smith and Circle, 1972; Zhu et al., 1996; Zilic et al., 2010; Pathiratne et al., 2015) pea germplasm resources and mutant materials with contrasting low and high LOX activity are limited, and no elite materials with low LOX and volatile profiles exist. A natural *LOX2* mutant was identified in a wild pea (*Pisum fulvum*), following a screen of diverse *Pisum* germplasm, and the mutant phenotype was introduced into the *P. sativum* cv. Birte by repeated backcrossing (Forster et al., 1999). However, this cultivar is not generally suitable agronomically for cultivation as a low LOX pea variety. Traditional breeding methods using this mutation entail repeated crossing, genotyping, and phenotyping, which are long and expensive processes to create an elite pea variety with low LOX activity and an improved flavor profile through hybridization.

In contrast, genome editing allows for the regular generation of mutations at preselected genomic loci in plants and could be used to facilitate the improvement of the flavor profile in seeds. In this work, we planned to generate null alleles (knockouts) for the most relevant LOX isoform in pea (Forster et al., 1999) using CRISPR constructs and transformation of elite yellow pea lines. Recent advancements in the deployment of CRISPR/Cas9 for genome editing in legumes was reviewed by Bhowmik et al. (2021). Soybean was the first legume crop targeted for genome editing with CRISPR/Cas technology. Several endogenous soybean genes (*GmLOX1, GmLOX2, GmLOX3*, *GmFT2, GmE1, GmFEI2*, and *GmSHR*) have been edited using the CRISPR/Cas9 method for improving traits such as beany flavor, flowering time, nodulation, and abiotic stress tolerance (Cai et al., 2018; Han et al., 2019; Wang et al., 2020). CRISPR/Cas9-mediated gene editing has also been successfully used in cowpea (Ji et al., 2019) and chickpeas (Badhan et al., 2021) for enhancing symbiotic nitrogen fixation and drought stress tolerance. These examples suggest that application of the CRISPR/Cas9 system for editing LOX gene(s) in elite yellow pea lines could provide improvement of the flavor profile in seeds as an alternative and sustainable approach to thermal treatment.

Therefore, the objectives of this study were two-fold: 1) To generate knockout LOX transgenic peas using a gene editing approach; and 2) To investigate the efficacy of this approach on improving the flavor profile of the transgenic peas.

## 2 Results

### 2.1 Generation of *pslox2* mutants and genotyping mutant lines

**Figure 1:**
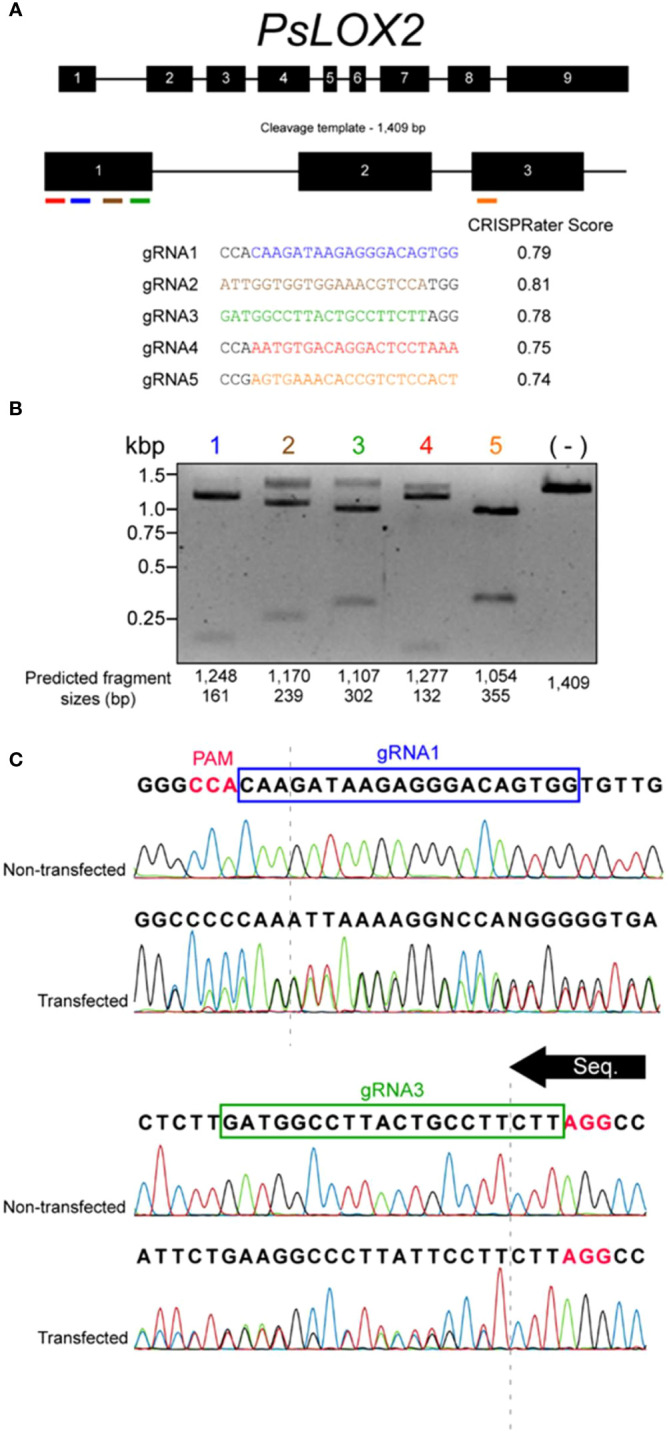
gRNA selection and protoplast assessment of *PsLOX2* CRISPR vector. **(A)** *PsLOX2* and location of candidate gRNA sites. Exons are represented by black boxes, introns are represented by black lines, and gRNA candidate sites are represented by colored lines. **(B)** *In vitro* cleavage assay of a PCR generated *PsLOX2* cleavage template incubated with recombinant Cas9 and *in vitro* transcribed gRNAs. (-) represents a reaction with no gRNA added. **(C)** Sanger sequencing chromatograms of the *PsLOX2* locus PCR amplified from protoplasts transfected with the *PsLOX2* CRISPR vector. The sequencing reaction occurred from the gRNA3 site to the gRNA1 site. The dashed lines represent the location of the DSB of the gRNA sites; Protospacer adjacent motif (PAM) sites are indicated in pink font.

*PsLOX2* was amplified from the pea cultivar CDC Spectrum (Warkentin et al., 2017) and analyzed using the web application CCTop (https://crispr.cos.uni-heidelberg.de; Stemmer et al., 2015) to design gRNAs. The five gRNA sequences with the highest CRISPRater scores (Labuhn et al., 2018) located in the first three exons of *PsLOX2* (**Figure 1A**) were selected. The cleavage ability of the gRNA candidate sequences was assessed with an *in vitro* Cas9 cleavage assay (**Figure 1B**). In this assay gRNAs 1 and 5 showed the greatest activity, cleaving almost all of the cleavage template. gRNAs 2, 3, and 4 were also able to cleave the template but to a lesser extent than gRNA 1 or 5. To maximize the probability of an edit we planned to express three gRNAs under the control of a single cauliflower mosaic virus 35S (35S) promoter using a tRNA-gRNA array (Xie et al., 2015). gRNAs 1, 3, and 5 were selected for cloning. However, we were unable to clone a single gRNA expression cassette expressing all three gRNAs. Instead, we were successful in producing a cassette containing only gRNAs 1 and 3. To expedite this project the gRNA expression cassette containing only gRNA1 and 3 was added to a binary vector which expressed Cas9 under the 35S promoter creating the *PsLOX2* CRISPR vector.

**Figure 2:**
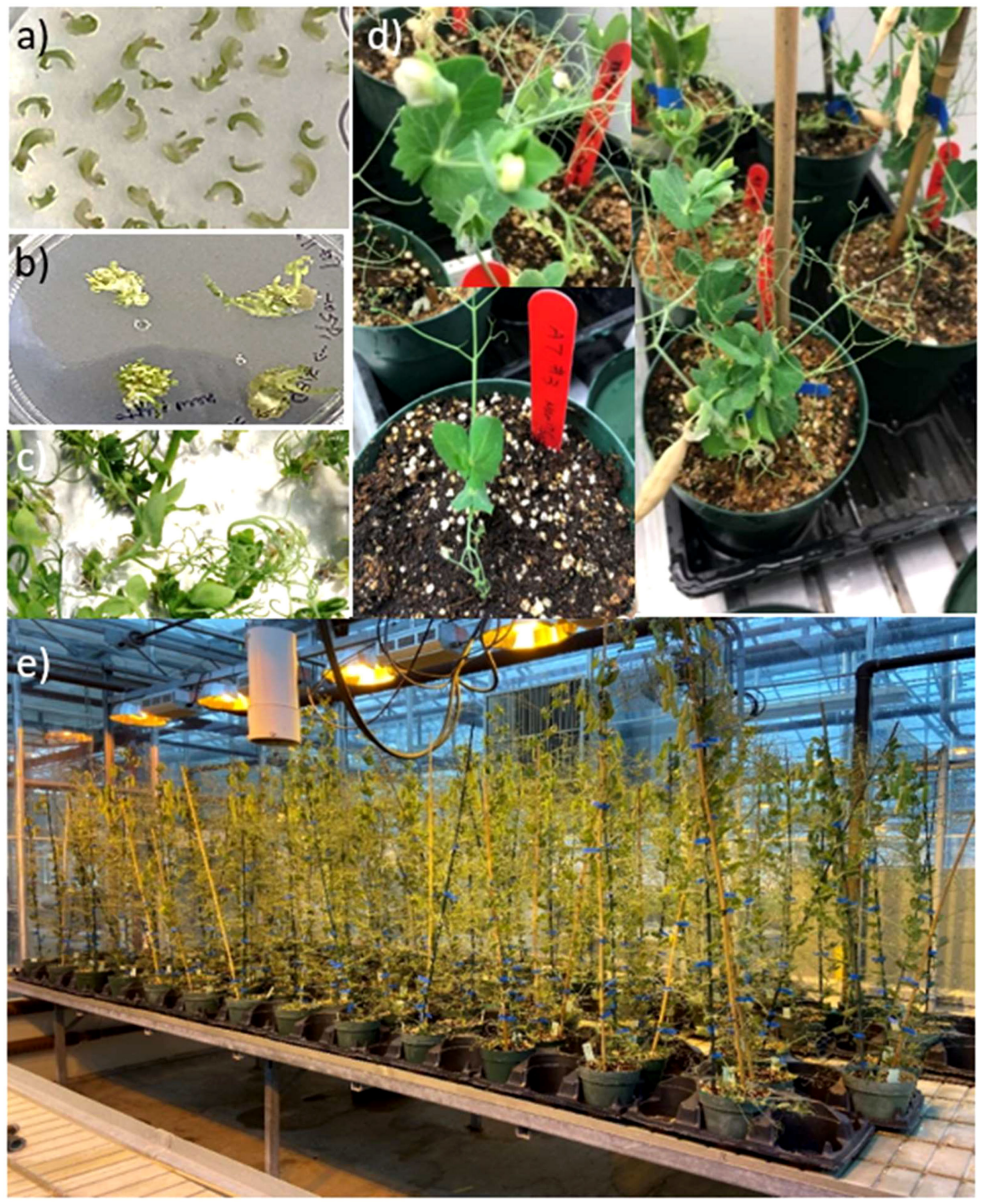
*Agrobacterium* mediated pea transformation and plant regeneration. **(A)** Slices of embryo axes after 4 days of co-cultivation with *Agrobacterium*. **(B)** Callus induction and shoot regeneration. **(C)** Explants at the end of a shoot induction phase. **(D)** Rooted putative transformant in soil. **(E)** Advancing T_2_ *PsLOX2* edited pea lines in the green house.

It takes 6–8 months to generate stable transgenic pea lines, making it time consuming to test CRISPR vectors in stable transgenic peas. To save substantial amounts of time and resources we assessed the *in planta* efficacy of the *PsLOX2* CRISPR vector using mesophyll protoplasts transfection (Pandey et al., 2022) before initiating stable transformation experiments. Protoplasts were generated from pea leaves and transfected with the *PsLOX2* vector. The *PsLOX2* target locus was PCR amplified from the transfected protoplasts and Sanger sequenced. Mixed signals in the sequencing chromatograms at the double stranded break (DSB) site of gRNA3 were interpreted as evidence of editing in the transfected protoplasts (**Figure 1C**). Based on this evidence, the *PsLOX2* CRISPR vector was transformed into the *Agrobacterium tumefaciens* strain EHA105 and putative pea transformants were regenerated (**Figure 2**).

**Figure 3:**
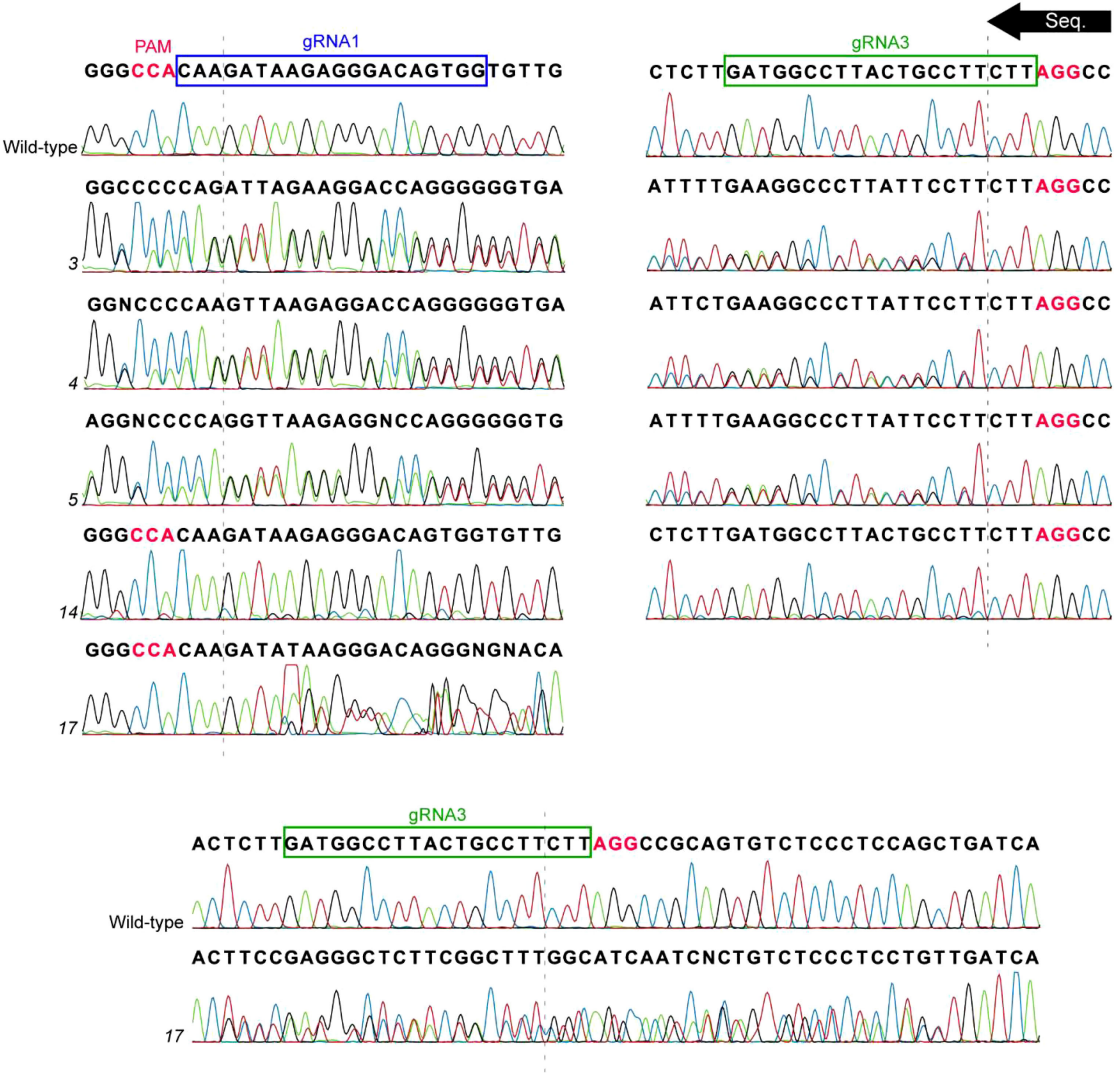
Sequencing data of T_1_ lines which showed evidence of mutation. The *PsLOX2* locus was PCR amplified and Sanger sequenced. The sequencing reaction occurred from the gRNA3 site to the gRNA1 site. The dashed lines represent the location of the DSB of the gRNA sites; Protospacer adjacent motif (PAM) sites are indicated in pink font.

A total of 19 plants were brought out of tissue culture, 17 of which were found to be transgenic. The gRNA1 and gRNA3 sites of *PsLOX2* were PCR amplified and analyzed for evidence of mutation using Sanger sequencing (**Table 1**; **Figure 3**). The resulting chromatograms were analyzed using Inference of CRISPR Edits analysis (ICE; https://ice.synthego.com/#/; Conant et al., 2022). Five lines (3, 4, 5, 14, and 17) showed secondary signals in their chromatograms at the DSB site of gRNA3 suggesting the presence of mutant alleles. ICE analysis determined that lines 3, 4, and 5 are heterozygous with a wild-type allele and a mutant allele with a 1 bp deletion at the gRNA3 site. The small secondary peak in the chromatogram of line 14 was not detected as a potential indel in the ICE analysis suggesting that potential mutant cells in this line are in the extreme minority. Interestingly, line 17 produced a secondary peak starting downstream of gRNA3 but did not extend for the entire length of the chromatogram (**Figure 3**). The ICE analysis of the target locus in line 17 detected a 44 bp deletion in 58% of indels and wild-type sequences in 30% of indels (the remaining 10% and 2% of indels were 14 and 16 bp deletions, respectively). This suggests that line 17 is heterozygous with one allele containing a large deletion of 44 bp at the gRNA3 site which explains why the double peak in the sequencing chromatogram is unable to extend to the full length of the wild-type allele’s sequencing chromatogram.

**Table 1:** Summary of genotyping data for T_1_ pea plants grown in soil.

Plant ID	Transgenic	gRNA 1		gRNA 3
1	Yes	WT		WT
2	Yes	WT		WT
3	Yes	WT		WT and T deletion
4	Yes	WT		WT and T deletion
5	Yes	WT		WT and T deletion
6	Yes	WT		WT
7	No	–		–
8	Yes	WT		WT
9	Yes	WT		WT
10	Yes	WT		WT
11	Yes	WT		WT
12	Yes	WT		WT
13	Yes	WT		WT
14	Yes	WT		WT
15	Yes	WT		WT
16	No	–		–
17	Yes	WT		WT and 44 bp deletion
18	Yes	WT		WT
19	Yes	WT		WT
WT	No	WT		WT

Genotyping assays were conducted using PCR from the T_1_ plants genomic DNA. Successful PCR using primers specific to *neomycin phosphotransferase II*, present on the T-DNA, was used to determine if plants were transgenic. Sanger sequencing of *PsLOX2* PCR products containing gRNA1 and gRNA3 sites was used to detect mutations. A single sequencing reaction reading from the gRNA3 to gRNA1 site was used. ICE analysis was used to determine the alleles present in mutated samples.

“–” indicates that no sequencing reaction was performed as the T_1_ plant was not transgenic and therefore could not be mutated.

**Figure 4:**
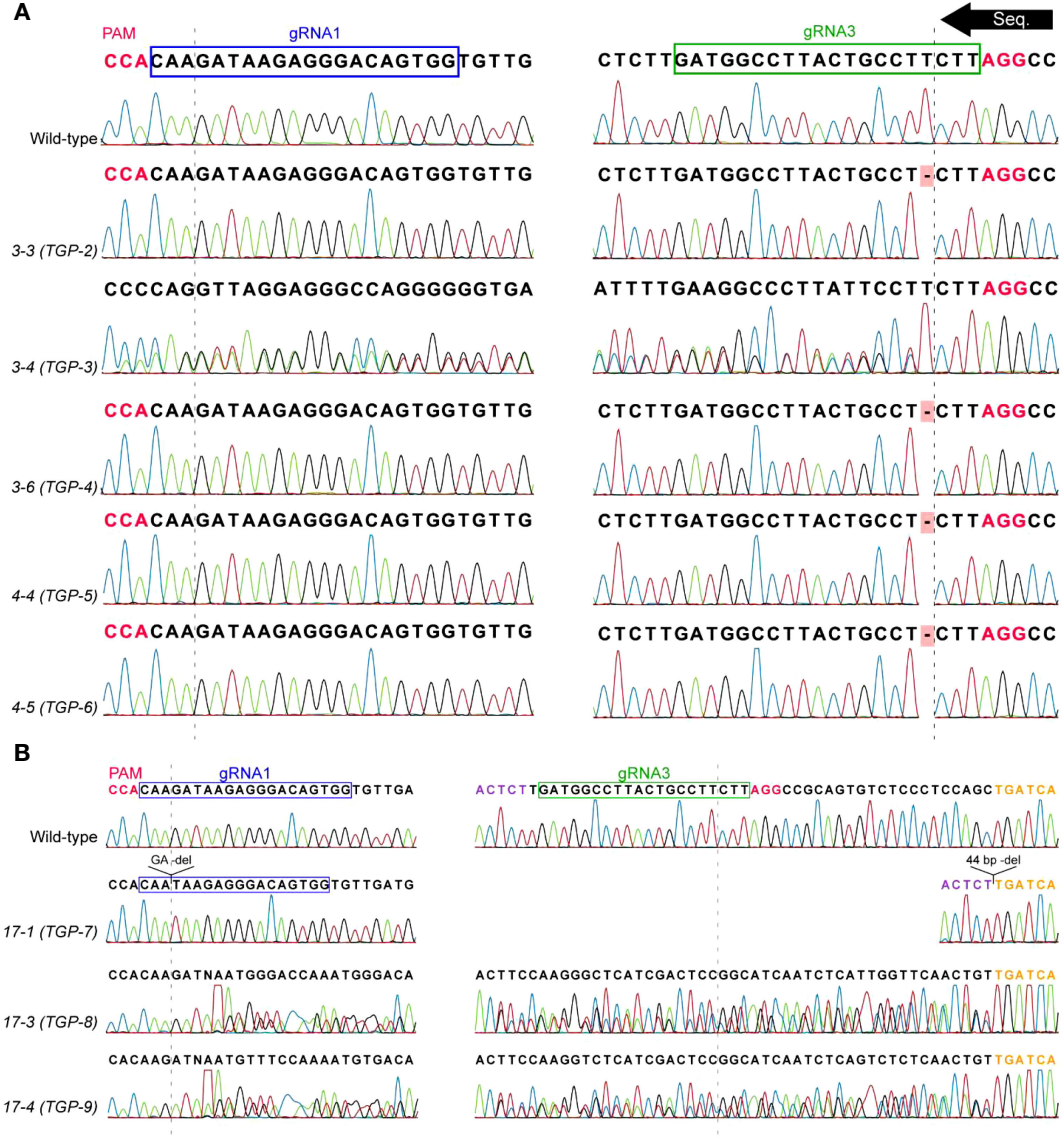
Sequencing data from T_2_ mutants used in LOX activity experiments. The *PsLOX2* locus was PCR amplified and Sanger sequenced. **(A)** T_2_ progeny of lines 3 and 4. **(B)** T_2_ progeny of line 17. Sequencing reactions occurred from the gRNA3 to gRNA1 site. The dashed lines represent the DSB site of the gRNA sites; Protospacer adjacent motif (PAM) sites are indicated in pink font; Purple and orange font indicate the flanking regions around the 44 bp deletion at the gRNA3 site of line 7-1.

To obtain homozygous mutant lines, three T_1_ lines (3, 4, and 17) which displayed strong evidence of mutation were advanced in the greenhouse to produce T_2_ plants (**Figure 2E**). Genomic DNA was extracted from the resulting T_2_ plants and analyzed as described for the T_1_ lines (**Table 2**; **Figure 4**). All T_2_ plants analyzed were transgenic. Two homozygous mutant T_2_ plants were identified in lines 3 and 4, all of which contained the same T deletion at the gRNA3 DSB site (**Figure 4A**). It is not uncommon for independent Cas9 cleavage events to result in the same indel mutations in independent transgenic lines (Bertier et al., 2018). Lines 3-4 and 4-3 were determined to be heterozygous containing a wild-type allele and T deletion allele. The identified T deletion causes a frameshift mutation resulting in an early stop codon after 66 amino acids (aa). *PsLOX2* is normally 863 aa and therefore this T deletion was assumed to eliminate *PsLOX2* activity.

**Table 2:** Summary of genotyping data for T_2_ pea plants.

T_1_ Line	Plant ID	Transgenic	gRNA1	gRNA3
3	3-1	Yes	WT	WT
	3-2	Yes	WT	WT
	3-3	Yes	WT	T deletion
	3-4	Yes	WT	WT and T deletion
	3-5	Yes	WT	WT
	3-6	Yes	WT	T deletion
	3-7	Yes	WT	WT
4	4-1	Yes	WT	WT
	4-2	Yes	WT	WT
	4-3	Yes	WT	WT and T deletion
	4-4	Yes	WT	T deletion
	4-5	Yes	WT	T deletion
	4-6	Yes	WT	WT
17	17-1	Yes	GA deletion	44 bp deletion
	17-2	Yes	WT	WT
	17-3	Yes	WT and GA deletion	WT and 44 bp deletion
	17-4	Yes	WT and GA deletion	WT and 44 bp deletion
WT		No	WT	WT

Genotyping assays were conducted as described in **Table 2** for T_1_ plants.

Three T_2_ plants from line 17 were found to be mutants, one of which was homozygous (**Table 2**; **Figure 4**). Line 17-1 was a homozygous mutant that had a GA deletion at the gRNA1 DSB site and a 44 bp deletion that completely removed the gRNA3 site. This GA deletion was not detected in the ICE analysis of the parental T_1_ line 17. Lines 17-3 and 17-4 had mixed signals in their chromatograms beginning 26 bp downstream of the gRNA3 DSB site, the same pattern as their T_1_ progenitor line. ICE analysis also indicated the predominant indels were 44 bp deletions and wild-type in lines 17-3 and 17-4. The genotypes of line 17’s T_2_ generation show classic 1:2:1 heterozygous Mendelian inheritance. This is strong evidence that the T_1_ progenitor line was heterozygous with one allele being wild-type and the other allele containing the mutations identified in line 17-1. The GA deletion at the gRNA1 site causes a frameshift mutation resulting in an early stop codon which dramatically reduces the size of the mutant protein to 22 aa, likely abolishing the function of *PsLOX2*.

### 2.2 LOX activity of *pslox2* mutant lines

To test the efficacy of the CRISPR gene edited *PsLOX2*, it was necessary to measure the LOX activity in the pea plants that have been edited and compare it to the LOX activity in unedited plants. Mutant lines 3-3, 3-4 (heterozygous), 3-6, 4-4, 4-5, 17-1, 17-3 (heterozygous), and 17-4 (heterozygous) were re-labelled as TGP-2 to -9, respectively, for LOX activity analysis (**Table 3**). In addition, the determination of the levels of linoleic acid and α-linolenic acid as the main known substrates of LOX enzymes in pulse flours, in both the edited and unedited peas was warranted. If the CRISPR editing of *PsLOX2* was effective in knocking out the gene, a decrease in LOX activity is expected followed by a significant increase in the levels of these key substrates. Finally, the determination of VOCs in the edited and unedited pea plants is also critical since these VOCs are directly involved in the generation of the beany aroma in cooked peas. Therefore, effective CRISPR editing of the LOX gene in peas would be expected to result in a significant decrease in beany flavor-related VOCs in the edited plants. Overall, by measuring the LOX activity, substrates, and VOCs in edited and unedited pea plants, the efficacy of the mutant *PsLOX2* lines may be assessed.

**Table 3:** Lipoxygenase specific activity (U/mg) and fatty acid composition (% of total fatty acid) in wild type (WT) and transgenic yellow pea (TGP) flours.

Enzyme Activity	F value (8, 18) †	Yellow Pea Samples								
		WT	TGP-2	TGP-3	TGP-4	TGP-5	TGP-6	TGP-7	TGP-8	TGP-9
Specific LOX activity (U/mg)	29.135 ***	170.18^a^ (14.64)	71.85^c^ (6.7)	94.75^bc^ (8.29)	89.04^bc^ (8.37)	93.86^bc^ (14.09)	103.2^b^ (6.39)	71.82^c^ (6.7)	100.6^b^ (5.58)	86.54^bc^ (8.90)
Fatty Acid Composition	F value (8, 18)	WT	TGP-2	TGP-3	TGP-4	TGP-5	TGP-6	TGP-7	TGP-8	TGP-9
Myristic acid (C14:0)	1.101 NS	0.23 (0.13)	0.35 (0.2)	0.23 (0.02)	0.17 (0.01)	0.17 (0.05)	0.18 (0.02)	0.18 (0.02)	0.18 (0.00)	0.35 (0.16)
Pentadecanoic acid (C:15)	11.770 ***	0.11^c^ (0.01)	0.14^ab^ (0.005)	0.15^a^ (0.005)	0.15^a^ (0.011)	0.14^ab^ (0.005)	0.15^a^ (0.007)	0.13^b^ (0.02)	0.14^ab^ (0.04)	0.15^ab^ (0.02)
Palmitic acid (16:0)	1.571 NS	11.53 (1.20)	11.39 (0.64)	11.42 (0.44)	11.62 (0.50)	10.88 (0.48)	11.47 (0.59)	10.42 (0.16)	10.81 (0.40)	11.59 (0.20)
Palmitoleic acid (C16:1)	4.556 *	0.070^a^ (0.006)	0.063^ab^ (0.010)	0.063^ab^ (0.005)	0.060^abc^ (0.003)	0.062^abc^ (0.003)	0.062^abc^ (0.002)	0.058^abc^ (0.008)	0.056^bc^ (0.001)	0.054^c^ (0.002)
Margaric acid (C17:0)	2.104 NS	0.17 (0.02)	0.19 (0.01)	0.19 (0.01)	0.19 (0.01)	0.18 (0.01)	0.18 (0.01)	0.18 (0.00)	0.19 (0.00)	0.20 (0.00)
Stearic acid (C 18:0)	6.700 ***	4.05^a^ (0.37)	3.55^b^ (0.10)	3.72^ab^ (0.14)	3.56^b^ (0.06)	3.39^b^ (0.05)	3.47^b^ (0.05)	3.42^b^ (0.03)	3.61^b^ (0.01)	3.36^b^ (0.06)
Oleic acid (C18:1) n-9	143.257 ***	27.83^e^ (0.88)	28.40^de^ (0.50)	32.89^a^ (0.13)	30.63^b^ (0.29)	30.26^bc^ (0.33)	29.39^cd^ (0.40)	25.94^f^ (0.13)	25.46^f^ (0.41)	23.44^g^ (0.20)
Linoleic acid (C18:2)	195.731 ***	45.25^c^ (0.39)	45.66^c^ (0.70)	42.27^e^ (0.26)	44.47^d^ (0.2)	45.35^cd^ (0.08)	45.4^cd^ (0.01)	49.05^b^ (0.05)	49.10^b^ (0.24)	50.50^a^ (0.38)
α-Linolenic acid (C18:3) n-3	27.181 ***	7.74^c^ (0.26)	7.38^cd^ (0.2)	6.48^e^ (0.4)	7.08^de^ (0.13)	7.56^cd^ (0.10)	7.67^cd^ (0.14)	8.61^a^ (0.03)	8.42^ab^ (0.17)	7.96^bc^ (0.07)
Arachidic acid (C20:0)	5.232 **	0.38^a^ (0.01)	0.366^ab^ (0.013)	0.370^ab^ (0.004)	0.351^b^ (0.003)	0.345^b^ (0.005)	0.371^ab^ (0.009)	0.36^ab^ (0.01)	0.37^ab^ (0.01)	0.34^b^ (0.01)
Behenic acid (C22:0)	26.637 ***	0.23^a^ (0.01)	0.156^b^ (0.002)	0.156^b^ (0.003)	0.146^b^ (0.003)	0.138^b^ (0.003)	0.147^b^ (0.003)	0.14^b^ (0.01)	0.13^b^ (0.02)	0.14^b^ (0.01)
Erucic acid (C22:1)	1.253 NS	0.05 (0.01)	0.044 (0.01)	0.061 (0.01)	0.062 (0.01)	0.053 (0.01)	0.060 (0.01)	0.05 (0.02)	0.04 (0.01)	0.05 (0.01)
Lignoceric acid (C24:0)	1.803 NS	0.38 (0.02)	0.35 (0.07)	0.35 (0.02)	033 (0.02)	0.32 (0.02)	0.32 (0.03)	0.32 (0.01)	0.32 (0.04)	0.31 (0.01)
Others ^♣^	4.203 **	1.98^a^ (0.26)	1.90^ab^ (0.70)	1.90^ab^ (0.70)	1.59^ab^ (0.20)	1.13^ab^ (0.04)	1.10^b^ (0.02)	1.08^ab^ (0.01)	1.13^ab^ (0.01)	1.06^ab^ (0.08)
∑ SFA	1.983 NS	17.25 (1.68)	17.03 (1.36)	16.80 (0.69)	16.57 (0.56)	15.60 (0.55)	16.33 (0.68)	15.20 (0.18)	15.78 (0.48)	16.94 (0.66)
∑ MUFA	132.557 ***	28.42^d^ (0.91)	28.96^cd^ (0.53)	33.51^a^ (0.11)	31.17^b^ (0.29)	30.82 ^b^ (0.35)	29.92 ^bc^ (0.45)	26.48^e^ (0.13)	25.98^e^ (0.42)	23.95^f^ (0.23)
∑ PUFA	119.466 ***	53.54^b^ (0.75)	53.36^b^ (0.91)	49.07^d^ (0.68)	51.64^c^ (0.24)	52.96^bc^ (0.19)	53.13^b^ (0.25)	57.70^b^ (0.06)	57.64^b^ (0.07)	58.56^b^ (0.45)
PUFA/SFA	6.174 ***	3.13^bc^ (0.36)	3.14^bc^ (0.30)	2.92^c^ (0.15)	3.11^bc^ (0.12)	3.39^ab^ (0.13)	3.25^ab^ (0.14)	3.80^a^ (0.05)	3.65^ab^ (0.11)	3.46^ab^ (0.16)
ω-6/ω-3	7.626 ***	5.67^c^ (0.22)	5.97^bc^ (0.14)	6.51^a^ (0.42)	6.25^ab^ (0.09)	5.98^bc^ (0.06)	5.90^bc^ (0.09)	5.68^c^ (0.02)	5.82^bc^ (0.15)	6.32^ab^ (0.04)

• Mean values (followed in brackets by the standard deviation) within the same row with the same letter are not significantly different when a probability level of 0.05 is applied. All analyses were conducted in three replicates (n = 3).

• MUFA, monounsaturated fatty acids; PUFA, polyunsaturated fatty acids; SFA, saturated fatty acids; ω-3, omega-3 fatty acids; ω-6, omega-6 fatty acids.

• Wild Type (WT), Transgenic 2 to 9; (TGP-2 to TGP-9).

• NS, not significant p ≥ 0.05; *p < 0.05; **p < 0.01; ***p < 0.001.

• **†**Numbers in the brackets reflect the degrees of freedom: F-value (dfbetween, dfwithin).

• ♣ Includes unidentified acids.

The activity of LOX was significantly reduced (P<0.05) in all transgenic peas (TGP-2 to -9) including the heterozygous lines (where the WT allele would be expected to be dominant) (**Table 3**). Among the TGP samples, TGP-2 and TGP-7 had the lowest LOX activity (71.8 U/mg) and TGP-6 had the highest (103.2 U/mg). This reflects ~58% and ~40% lower activity of LOX compared to WT samples for TGP-2 and -7, and TGP-6, respectively.

### 2.3 FA profile of *pslox2* mutant lines

It was also important to investigate the effect of this significant decrease in LOX activity obtained for transgenic pea samples on the levels of FAs since some essential PUFAs have been previously identified as substrates of LOX enzymes in various types of pulses (Murray et al., 1976; Sessa and Rackis, 1977; Lampi et al., 2020). The FA composition of the WT and transgenic pea flours is shown in **Table 3**. All values are reported as a percentage of total FAs detected using GC-FID. The FA content of the WT peas was composed primarily of unsaturated fatty acids of which 53.54% were PUFA and 28.42% were monounsaturated fatty acids (MUFA). Saturated fatty acids (SFAs) comprised 17.24% of the total lipid content. Several major FAs were present including linoleic acid (C18:2) (45.25%), followed by oleic acid (C18:1, 27.83%), palmitic acid (C16:0, 11.53%), *α*-linolenic acid (C18:3, 7.74%), and stearic acid (18:0, 4.05%). The SFA levels were not affected by CRISPR-mediated changes of *PsLOX2* while both PUFAs and MUFAs were significantly affected in all transgenic peas but only PUFAs were found at higher percentages in lines TGP-7, -8, and -9 compared to WT peas. Of particular importance, the specific substrates of LOX enzymes, linoleic acid and *α*-linolenic acid were both found at higher concentrations in TGP -7, -8, and -9. The transgenic peas had a significantly increased range covering from 8.4 to 11.6% in their linoleic acid content and an increased range from 2.8 to 11.2% in their *α*-linolenic acid content. One of the important indices used for evaluating the nutritional value of dietary foods, the *ω*-6/*ω*-3 ratio was also reported in **Table 3**. The ratio of *ω*-6/*ω*-3 in WT pea flours was 5.67 and only significantly increased in TGP–3, -4, and -9 samples by ~11–14%.

### 2.4 VOC composition of *pslox2* mutant lines

A list of 11 VOCs collected using SPME extraction is provided in **Table 4**. Among these, 10 were significantly decreased in transgenic pea flours compared to WT pea flour. Identification of these 11 VOCs was performed by matching the detected mass spectra and LRI values of the VOCs with the values reported by the NIST library (2017, version 2.3). The probable origin and sensory attributes of each compound are also reported for further confirmation for each VOC. The majority of detected compounds in these pea extracts were aldehydes and alcohols.

**Table 4:** Concentrations of selected volatile compounds (μg/100g of flour) in wild type (WT) and transgenic yellow pea (TGP) flours.

Volatile Organic Compound (VOC)	Probable origin	Ref	Odor description	LRI^♠^	F Value (8, 33)^†^	WT	TGP-2	TGP-3	TGP-4	TGP-5	TGP-6	TGP-7	TGP-8	TGP-9
Hexanal	Linoleic acid	(Eriksson, 1975) (Murray et al., 1976) (Jelen and Wasowicz, 2011) (Murat et al., 2013) (Mandal et al., 2014) (Lampi et al., 2020) (Belitz et al., 2009)	Green, Grassy	881	10.574 ***	25.321^a^ (9.213)	9.546^bc^ (3.83)	19.860^ab^ (5.90)	7.360^bc^ (1.93)	7.090^bc^ (1.16)	7.150^bc^ (0.96)	5.736^bc^ (0.440)	13.384^bc^ (3.550)	11.634^bc^ (2.956)
2-Hexenal	Linoleic acid Linolenic acid	(Murray et al., 1976) (Sessa & Rackis, 1977) (Mandal et al., 2014) (Jelen and Wasowicz, 2011) (Lampi et al., 2020)	Green, Leafy	958	19.613 ***	8.910^a^ (3.621)	0.648^b^ (0.24)	6.462^a^ (1.35)	0.672^b^ (0.25)	0.476^b^ (0.21)	0.522^b^ (0.13)	0.353^b^ (0.033)	1.757^b^ (0.764)	2.543^b^ (0.792)
1-Hexanol	Linoleic acid	(Murray et al., 1976) (Jelen and Wasowicz, 2011) (Matoba et al., 1989)	Green, Herbacious	978	0.680 NS	2.672^a^ (1.689)	1.866^a^ (0.60)	2.032^a^ (0.43)	1.686^a^ (0.46)	2.126^a^ (0.16)	2.061^a^ (0.09)	2.053^a^ (0.208)	2.102^a^ (0.215)	1.851^a^ (0.748)
Heptanal	Linoleic and oleic acids Linolenic acid	(Murray et al., 1976) (Belitz et al., 2009) (Lampi et al., 2020) (Sessa & Rackis, 1977)	Green, Fatty, Pungent	984	4.155 **	2.216^a^ (1.179)	1.255^ab^ (0.47)	1.376^ab^ (0.26)	0.769^b^ (0.11)	0.819^b^ (0.13)	0.927^b^ (0.10)	0.731^b^ (0.043)	1.210^ab^ (0.497)	0.977^b^ (0.173)
(E)-2-Heptenal	Linoleic acid α-Linolenic acid	(Hoffmann, 1962) (Eriksson, 1975) (Sessa and Rackis, 1977) (Belitz et al., 2009) (Lampi et al., 2020) (Murray et al., 1976) (Belitz et al., 2009)	Beany, Pungent, Green	1064	8.583 ***	7.785^a^ (3.309)	2.637^b^ (0.55)	3.429^b^ (0.32)	1.913^b^ (0.50)	1.767^b^ (0.41)	2.069^b^ (0.26)	1.855^b^ (0.106)	2.918^b^ (1.601)	3.195^b^ (1.298)
1-Octen-3-ol	Linoleic acid *α*-Linolenic and Linoleic acids	(Lampi et al., 2020) (Hoffmann, 1962) (Khrisanapant et al., 2019) (Murray et al., 1976) (Zhang et al., 2020) (Lumen et al., 1978) (Mandal et al., 2014)	Earthy, Mushroom, Vegetable	1082	5.590 **	7.779^a^ (3.80)	3.114^b^ (1.02)	3.768^b^ (0.52)	2.694^b^ (0.48)	2.717^b^ (0.30)	3.085^b^ (0.74)	2.717^b^ (0.39)	3.646^ab^ (0.74)	3.140^b^ (0.59)
Furan-2-Pentyl	Linoleic acid	(Min and Boff, 2002)	Green	990	4.921 *	42.657^a^ (21.406)	16.205^b^ (5.70)	18.353^ab^ (5.54)	9.672^b^ (2.32)	11.844^b^ (1.51)	13.021^b^ (2.74)	11.367^b^ (2.697)	22.204^ab^ (12.743)	24.419^ab^ (12.283)
Octanal	Oleic acid Linoleic acid	(Murray et al., 1976) (Belitz et al., 2009) (Belitz et al., 2009)	Green	1088	2.796 *	4.423^a^ (3.015)	1.781^b^ (0.85)	1.343^b^ (0.34)	0.784^b^ (0.39)	0.923^b^ (0.17)	1.348^b^ (0.26)	1.109^b^ (0.247)	1.477^b^ (0.622)	1.289^b^ (0.544)
(E,E)-2,4-Heptadienal	*α*-Linolenic acid	(Murray et al., 1976) (Lampi et al., 2020)	Rancid hazelnut, Brown beany	1138	8.192 **	3.897^a^ (1.804)	1.130^b^ (0.18)	1.479^b^ (0.05)	0.810^b^ (0.20)	0.616^b^ (0.28)	0.877^b^ (0.10)	0.786^b^ (0.106)	1.640^b^ (0.643)	1.739^b^ (0.853)
(E)-2-Octenal	Linoleic acid	(Murray et al., 1976) (Sessa & Rackis, 1977) (Belitz et al., 2009) (Murat et al., 2013) (Lampi et al., 2020)	Green, Leafy, Brown, Pea, Vegetable	1168	7.885 ***	3.678^a^ (1.595)	1.139^b^ (0.36)	1.547^b^ (0.25)	0.754^b^ (0.26)	0.685^b^ (0.22)	0.900^b^ (0.15)	0.739^b^ (0.153)	1.667^b^ (0.798)	1.446^b^ (0.909)
(E,E)-2,4-Nonadienal	Linoleic acid α-Linolenic acid	(Murray et al., 1976) (Sessa & Rackis, 1977) (Jelen and Wasowicz, 2011)	Green, Grassy, Sweat	1348	6.033 **	1.723^a^ (0.908)	0.636^b^ (0.20)	0.737^ab^ (0.04)	0.352^b^ (0.09)	0.289^b^ (0.11)	0.403^b^ (0.08)	0.324^b^ (0.086)	0.887^ab^ (0.452)	0.685^b^ (0.390)

• Mean values (followed in brackets by the standard deviation) within the same row with the same letter are not significantly different when a probability level of 0.05 is applied. All analyses were conducted in triplicate (n=3) except for Wild Type (n=7).

• Wild Type (WT), Transgenic 2 to 9; (TGP-2 to TGP-9).

• NS, not significant p ≥ 0.05; *p < 0.05; **p < 0.01; ***p < 0.001.

• **^♠^** Linear Retention Index (LRI): calculated using the retention time obtained for a series of n-alkane (C8-C20, 40 μg/ml hexane) as described previously (Aliani and Farmer, 2005).

• †Numbers in the brackets reflect the degrees of freedom: F-value (dfbetween, dfwithin).

A significant decrease in the concentration of known key VOCs with unpleasant aromas (i.e., beany, green, grassy) such as hexanal, 2-hexenal, heptanal, (E)-2-heptenal, 1-octen-3-ol, furan-2-pentyl, (E,E)-2,4-heptadienal, (E)-2 octenal, and (E,E) 2,4-nonadienal was observed in at least one of the eight transgenic pea samples presented in our study when compared to WT untreated pea flour. Across eight of the ten significantly reduced VOCs (hexanal, 2-hexanal, (E)-2-heptanal, 1-octen-3-ol, furan-2-pentyl, (E,E)-2,4-heptadienal, (E)-2-octenal, and (E,E)-2,-4-nonadienal) the homozygous mutant lines TGP-2, -4, -5, -6, and -7 seem to have a more severe reduction than the heterozygous mutant lines TGP-3, -8, and -9. The decrease in the aforementioned VOCs in transgenic lines is reasonable given the reduction in LOX activity.

**Figure 5:**
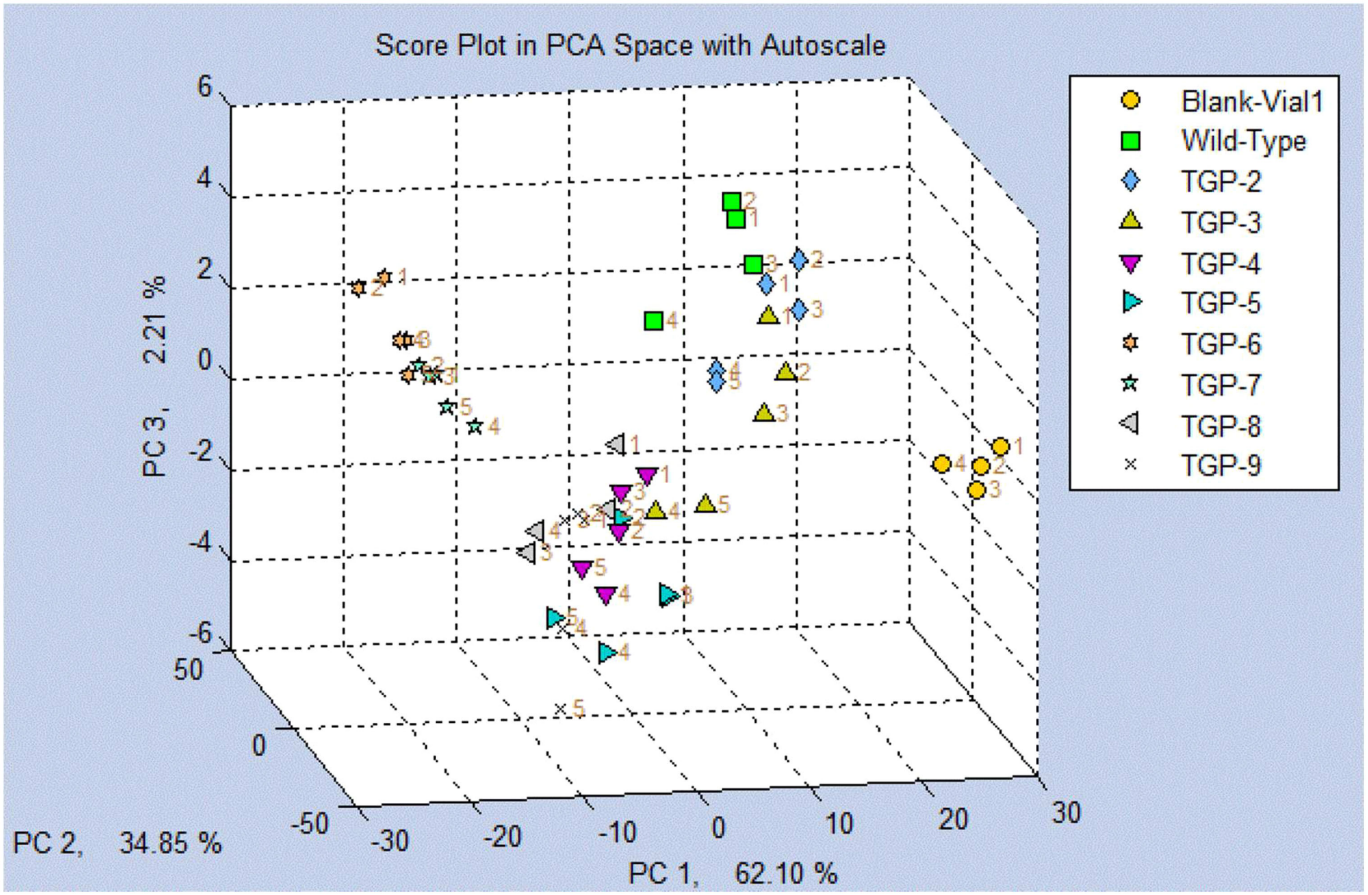
Score plot using principal component analysis (PCA) for Wild Type (WT) and all transgenic yellow pea flours (TGP-2 to -9) by SENSIGENT MSEM 160 Portable Odor and Chemical Monitor System.

A PCA score plot was generated based on all the VOCs collected from the headspace of WT and transgenic pea samples compared to a blank (air) (**Figure 5**). The resulting plot shows a clear separation of clusters from the blank sample and a separation of the TGP lines clusters from the WT line cluster. TGP-4 to -9 were further separated from the TGP-2 and -3 lines. Overall, more than 95% of the cluster separation was explained by the first two PC axes with 62.10% and 34.85% for PC1 and PC2 axes, respectively.

Multiple regression analysis showed that two VOCs, heptanal and (E,E)-2,4-heptadienal, were mostly correlated with LOX activity in pea flours. The standardized beta coefficient values that compare the strength of the effect of individual predictors (independent variable) to LOX activity in the investigated model were as follows: 0.946 (*p* = 0.003) for (E,E)-2,4-heptadienal; 0.055 (*p* = 0.003) for heptanal. The stepwise model was significant (F1,2 = 3376.572, *p* = 0.003) with the adjusted *R*^2^ value of 1.00.

## 3 Discussion

LOX activity in yellow peas causes the production of VOCs from PUFAs which are associated with unpleasant aromas in pea flour. While other approaches like micronization and RevTech heat treatment have been demonstrated to diminish the unpleasant aromas of various pulse flours by totally or partially deactivating LOX activity (Shariati-Ievari et al., 2016; Fahmi et al., 2021), these methods lack selectivity and can impact other enzymes in pulse flours. The utilization of CRISPR/Cas9 technology presents an excellent option to target *PsLOX* genes precisely for mutation thereby reducing LOX activity in pea flour and reducing the production of undesirable VOCs.

The application of CRISPR/Cas9 as a tool for rapid and efficient genome modification and accelerated crop improvement has been successful for soybean genome editing (Cai et al., 2015; Jacobs et al., 2015; Li et al., 2015; Michno et al., 2015; Sun et al., 2015; Du et al., 2016; Curtin et al., 2018; Kanazashi et al., 2018). The recently available sequence of the pea genome (Kreplak et al., 2019) has provided a valuable tool for reverse genetics approaches such as CRISPR/Cas9 to realize the agricultural productivity and nutritional value of this crop. Li et al. (2023) demonstrated CRISPR/Cas9 mutation of *phytoene desaturase* in peas. However, to the best of our knowledge, this is the first report of the use of CRISPR/Cas9 to generate mutant pea lines with improved flavor traits.

Soybean lines with mutations of all three *GmLOX* isogenes (*GmLOX1/2/3*) have been identified (Hildebrand and Hymowitz, 1982; Kitamura et al., 1983; Davies et al., 1987). Lines with null mutations of *GmLOX2* were particularly valuable as they were determined to have improved flavor in human sensory tests whereas lines with mutations in *GmLOX1/3* did not have improvments over the wild-type (Davies et al., 1987). Importantly, disease resistance and seed yield were not negatively impacted by the *GmLOX2* null mutation (Pfeiffer et al., 1992). Similarly, mutants of *pslox2*, one of the two seed *PsLOX* isogenes, had no decrease in seed weight or field yield despite having reduced VOC levels (Forster et al., 1999). VOCs have been reported to have roles in a diverse set of plant metabolic pathways (Vancanneyt et al., 2001). However, the unique environmental conditions experienced by soybean and pea *LOX* mutants in agricultural fields can potentially explain their ability to combat this deficiency. The introgression of the identified *pslox2 i*nto elite cultivars is possible but will be a time intensive endeavor. In contrast recreating this trait in an elite cultivar is an obvious application for CRISPR/Cas9 mutagenesis as a simple single gene knockout can dramatically improve the flavor of the pea seeds.

To this end, we implemented a previously successful CRISPR/Cas9 editing system in the elite yellow pea cultivar CDC Spectrum and successfully generated eight transgenic *pslox2* mutant lines. Confirming significantly decreased LOX activity in pea flours generated from each of the eight transgenic lines was crucial. Although all transgenic lines had significantly (p<0.05) lower LOX activity, TGP-7 had the lowest LOX activity. Previous studies have demonstrated that heat treatments such as RevTech at 140°C with 0% and/or 10% moisture were effective means to decrease LOX activity in yellow peas by 43% and 89%, respectively (Fahmi et al., 2021). The *pslox2* mutants described here had similar reductions in LOX activity (39.4% to 57.8%) as the RevTech treatment but required no heating or further processing.

The FA compositions presented in **Table 3** are consistent with those previously reported in different varieties of peas, suggesting that the major FAs in pea flours are linoleic, oleic, palmitic, and α-linolenic acid, in decreasing order (Murcia and Rincón, 1992; Grela and Günter, 1995; Ryan et al., 2007; Villalobos Solis et al., 2013; Caprioli et al., 2016; Padhi et al., 2017; Khrisanapant et al., 2019; Fahmi et al., 2021). The overall FA profiles were not affected in transgenic pea samples. However, some essential PUFAs, linoleic and α-linolenic acid, were found at higher concentrations in some transgenic peas (TGP-7, -8, and -9). This is expected as LOX activity was reduced in the transgenic pea lines due to mutations in *PsLOX2* (**Table 3**). If this significant increase in the proportion of linoleic and α-linolenic acid is due to reduced LOX activity, a decrease in the key known VOCs generated from these PUFAs is to be expected. In line with this expectation, there were significantly lower concentrations of 10 out of 11 VOCs in the transgenic peas (**Table 4**). The difference among the TGPs tested here might relate to other interfering co-factors required for LOX activity.

The *ω*-6/*ω*-3 ratios reported here are consistent with previously reported values ranging from 4.4 to 6.3 (Grela and Günter, 1995; Ryan et al., 2007; Caprioli et al., 2016; Khrisanapant et al., 2019).

Identification and semi-quantification of VOCs associated with unpleasant aromas was achieved based on the comparison of mass spectra and LRI values of VOCs detected in WT and *pslox2* mutant pea flours with those reported in the NIST library (2017, version 2.3) (**Table 4**). For additional clarity, the likely fatty acid source and odor description of each VOC can be found in **Table 4**. Most VOCs identified in the pea seed extracts were aldehydes or alcohols. A significant decrease in all TGP lines was only seen for VOCs (E)-2-heptenal, octanal, (E,E)-2,-4-heptadienal, and (E)-2-octenal.

The standardized beta coefficient values [the measure of the contribution of the variables (all significant VOCs and FAs) to the model)] indicated the strength of the effect of each model predictor variable used on LOX activity. Overall, the LOX activity of pea samples was highly influenced by (E,E)-2,4-heptadienal and heptanal. Beta values were 0.946 and a p-value of 0.003 ((E,E)-2,4-heptadienal) and 0.055 and a p-value of 0.003 (heptanal). These findings suggest that (E,E)-2,4-heptadienal and heptanal are important VOC markers of LOX activity in pea flours, with (E,E)-2,4-heptadienal having a much stronger effect compared to heptanal. The stepwise model was significant with an F1,2 value of 3376.572 and a p-value of 0.003, indicating that the model is a good fit for the data. The adjusted R2 value of 1.00 suggests that the model explains 100% of the variance in LOX activity, which is a very strong result. These VOCs could help food developers to create suitable screening models to select pea flours and/or products made with them to predict unpleasant aroma.

This means that as the LOX activity increases the concentration of (E,E)-2,4-heptadienal increases. LOX activity also had a significant effect on heptanal, but its effect was much weaker, with a standardized beta coefficient of 0.055 and a p-value of 0.003. This suggests that as the LOX activity increases, the concentration of heptanal also increases, but the effect is much smaller compared to (E,E)-2,4-heptadienal.

Collecting volatiles using sensors typically involves using specialized devices or instruments that are designed to detect and measure the concentration or presence of volatile compounds in the air or other environments. These sensors can be based on various principles such as chemical reactions, physical properties, or electrical signals, depending on the type of volatile compound being targeted. The SENSIGENT MSEM 160 Portable Odor and Chemical Monitor System used in our study uses an array of 32 sensors to mimic the sense of smell for detecting volatiles from a wide range of chemical classes which makes them useful to screen food samples. These results show the overall VOC profile of the pea flours and not the individual VOCs that were identified using the GC-MS analysis outlined above. The score plot in principal component analysis (PCA) for WT and eight transgenic yellow pea flours (TGP-2 to -9) presented in **Figure 5** clearly shows the ability of this device to distinguish between CRISPR-mediated and nonmediated pea flours with more than 96% of the separation explained by the first two PCA axes.

The homozygous mutant lines TGP-2, -4, -5, -6, and -7 tended to have a more severe phenotype than the heterozygous mutant lines TGP-3, -8, and -9 across the parameters examined, LOX activity, FA composition, and VOC composition. The data suggest that *PsLOX2* shows semi-dominant inheritance as the heterozygous mutants have an intermediate phenotype between the homozygous mutant and WT lines. Although unexpected, a similar semi-dominant inheritance has been observed in tomato (*Solanum lycopersicum*) mutants of *suppressor of prosystemin-mediated responses8* (*spr8*) which is also a lipoxygenase (Yan et al., 2013).

**Figure 6:**
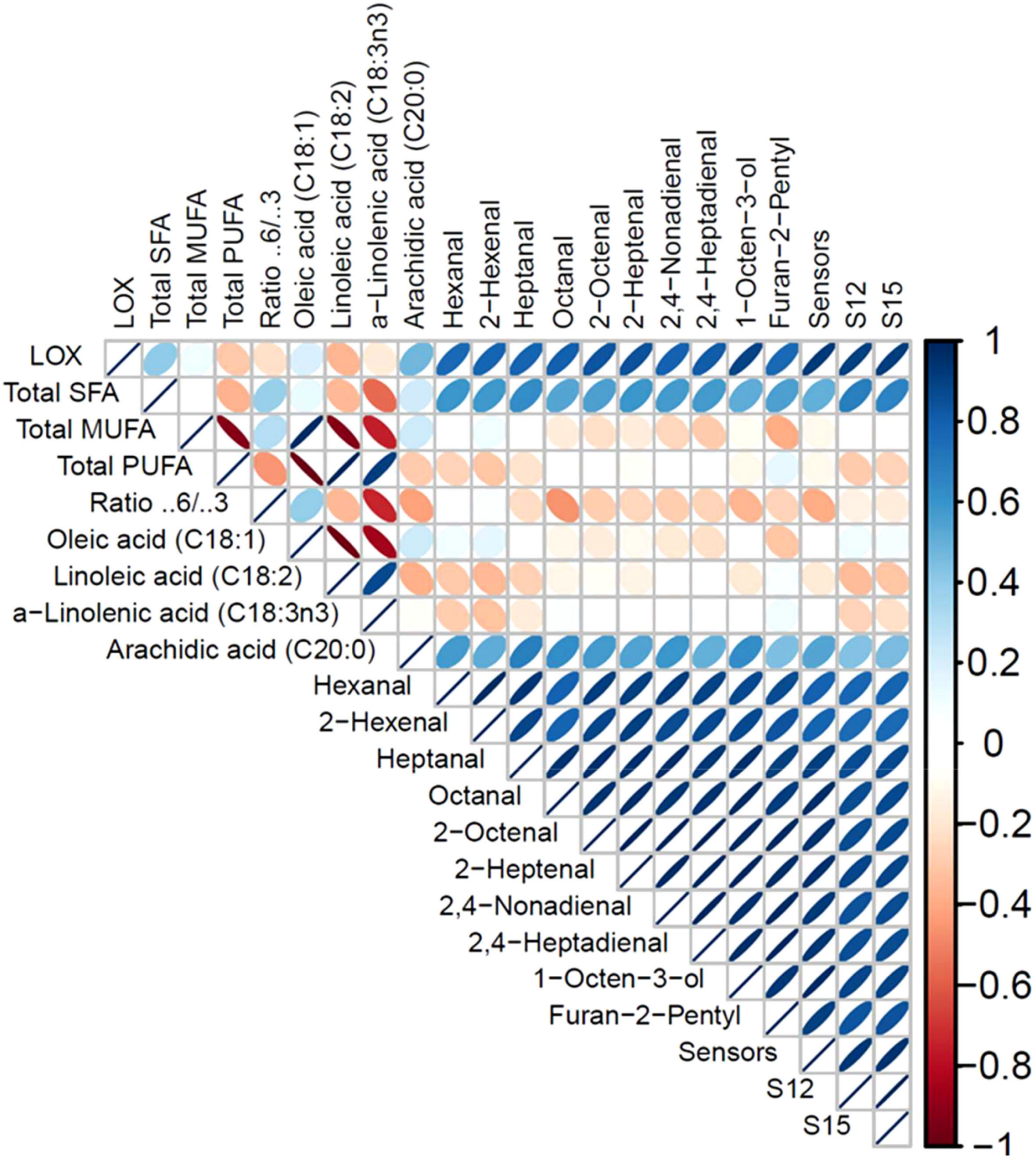
R Correlogram for LOX activity, selected fatty acids (FAs), and concentration of volatile organic compounds (VOCs) in Wild Type and all Transgenic Yellow Pea Flours (TGP-2 to -9). The correlogram is color-coded with the correlation scale indicated. Blue color shows a positive correlation and red color a negative correlation, as indicated by opposite directions of ellipses within each square. The width of the ellipse represents the strength of the correlation, perfect linear is just a line.

To visually examine the linear correlation between LOX activity and all significant measurements presented in **Tables 3** and **4**, a correlogram is provided in **Figure 6**. This graph presents a positive linear correlation between LOX and detected VOCs with the sensor responses. A negative correlation is obtained for total PUFAs, selective PUFAs (linoleic and α-linolenic acid), and also for the ω-6/ω-3 ratios.

**Figure 7:**
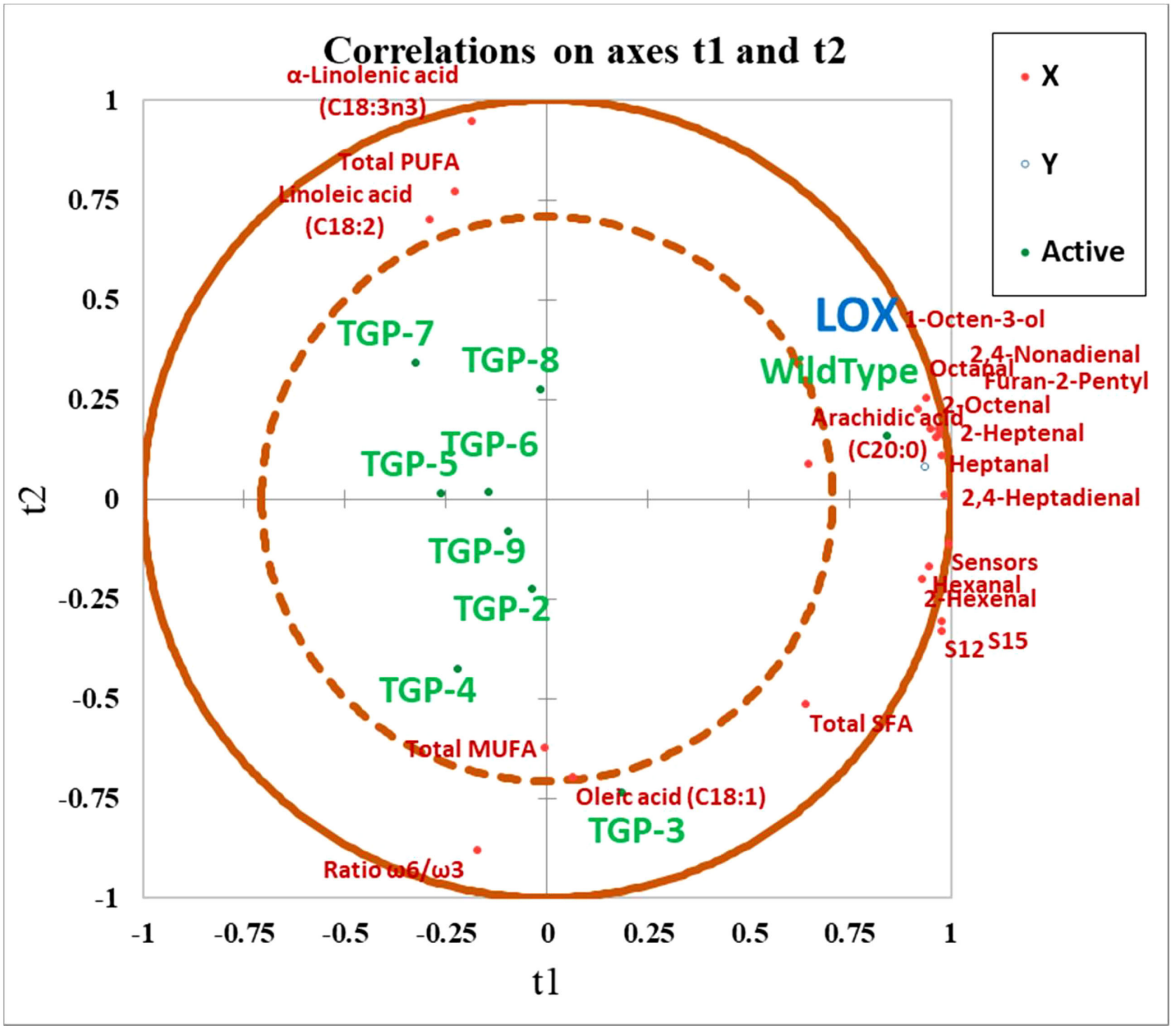
Partial least squares regression (PLS-R) plot for wild type and all transgenic yellow pea flours (TGP-2 to -9). Observations showing the correlation between *X* (t1) and *Y* (t2) variables where: *X* variables • = volatile organic compounds (VOCs) responsible for beany off-flavor, selected fatty acids, and E-nose sensor response; *Y* variable • = LOX activity observations; ⚬ = Wild Type, and transgenic yellow pea flours.

In addition, the overall correlations between selected VOCs, FAs, sensor responses, and LOX activity in WT and TGP lines are presented in the PLS-R plot presented in **Figure 7**. The eight TGP lines were clustered separately in different areas on the plot opposite to WT lines which were located in the quadrant close to the LOX activity, sensor responses, and also with the majority of VOCs responsible for unpleasant aromas. Overall, the plot depicts a positive association between the LOX activity and VOC formation and is a useful visual model to examine the significant results as a whole rather than discussing them one by one. It is also clear that as expected for a *LOX* mutant, the SFAs and MUFAs were not affected by CRISPR/Cas9 gene editing of the *PsLOX2* gene and these FAs were all found to be associated with WT lines.

## 4 Conclusions

The results presented in this study demonstrated a successful CRISPR-Cas9 mediated editing of the pea *PsLOX2* gene. The results obtained for LOX activity, the known substrates, and products of this enzyme confirmed that this mutation led to lower activity of LOX in all mutant pea lines with a significant decrease in several VOCs generated from some PUFAs. Among all the detected VOCs, (E,E)-2,4-heptadienal may be of particular interest for monitoring the success of CRISPR-Cas9 mediated editing of the pea *PsLOX2* gene.

The use of sensors to monitor the headspace of the pea flours in less than 3 minutes provided a rapid and sensitive tool for screening the VOCs profile of any transgenic crop which can accelerate breeding and gene discovery in pea and other important legume crop plants.

## 5 Materials and methods

### 5.1 Pea growth conditions

Yellow pea seeds of the elite genotype CDC Spectrum were obtained from the Crop Development Centre, University of Saskatchewan (Dr. Tom Warkentin’s pea breeding program). Plants were grown in a controlled environment chamber at 24°C with a 16 h photoperiod at 200 μmol quanta m^−2^ s^−1^.

### 5.2 Amplification of *PsLOX2* from cv. CDC Spectrum, identification and designing of gRNAs, and CRISPR/Cas9 vector construction

Primers (all primers are listed in **Table 5**) were designed based on the accession X78580 (GenBank) and were used to PCR amplify the first three exons of *PsLOX2* from the pea cv. CDC Spectrum (Warkentin et al., 2017). The resulting PCR product was cloned into the *EcoRV* site of pBluescript using Gibson assembly and sequenced using primers 3/4. The web-application CCTop (https://crispr.cos.uni-heidelberg.de; Stemmer et al., 2015) was then used to identify gRNA sites in this sequence. The five gRNA sites located in the first three exons with the highest CRISPRater scores (Labuhn et al., 2018) were selected for *in vitro* testing. CRISPRater is an algorithm which predicts the efficiency of a gRNA based on its sequence. *In vitro* tests were performed as described by Kwon et al. (2023). Briefly, DNA templates for *in vitro* transcription of gRNA candidates were produced using PCR with primers 5–9 and a universal reverse primer (10) from a tracrRNA template used in a previous study (Kwon et al., 2023; Invitrogen, Waltham, MA, USA). gRNAs were prepared using the HiScribe™ T7 Quick High Yield RNA Synthesis Kit (NEB, Ipswich, MA, USA). The cleavage template was prepared from the pBluescript clones using primers 3/4. Recombinant Cas9 was prepared as described by Kwon et al. (2023). The gRNA, cleavage template, and recombinant Cas9 were incubated at 37°C for 1 h and the resulting DNA fragments were analyzed using gel electrophoresis.

**Table 5:** List of primers used in this work.

No.	Sequence
1	GCTGCAGGAATTCGAT ATGTTTCCAAATGTGACAGGACTCC
2	CGGTATCGATAAGCTTGAT CTTGTATCTAACCTTTCCTCGTTGG
3	CACACAGGAAACAGCTATGA
4	CGTTGTAAAACGACGGCCAG
5	GAAATTAATACGACTCACTATAGGCCACTGTCCCTCTTATCTTG GTTTTAGAGCTAGAAA
6	GAAATTAATACGACTCACTATAGGATTGGTGGTGGAAACGTCCA GTTTTAGAGCTAGAAA
7	GAAATTAATACGACTCACTATAGGGATGGCCTTACTGCCTTCTT GTTTTAGAGCTAGAAA
8	GAAATTAATACGACTCACTATAGGTTTAGGAGTCCTGTCACATT GTTTTAGAGCTAGAAA
9	GAAATTAATACGACTCACTATAGGAGTGGAGACGGTGTTTCACT GTTTTAGAGCTAGAAA
10	AAAAAAGCACCGACTCGGTGC
11	AGGCTACCGCATAAGTCC
12	CCACTGTCCCTCTTATCTTG GTTTTAGAGCTAGAAATAGCAAGTTAAAATAAGGCTAG
13	AAGAAGGCAGTAAGGCCATC TGCACCAGCCGGGAATC
14	GATGGCCTTACTGCCTTCTT GTTTTAGAGCTAGAAATAGCAAGTTAAAATAAGGCTAG
15	ACTGGTGATTTTTGCGGACT TGCACCAGCCGGGAATC
16	TTCGATTCCCGGCTGGTGCA CCACTGTCCCTCTTATCTTG
17	AAGAAGGCAGTAAGGCCATC
18	TGAGAATTAAGGGAGTCACGTTATGACC
19	CGGCCATTTTCCACCATGATATT

Underlined sections are overhangs.

The *PsLOX2* CRISPR vector was prepared as described by Kwon et al. (2023). Briefly, gRNAs 1 and 3 were selected for cloning into the CRISPR vector based on the *in vitro* cleavage assay. To express both gRNAs simultaneously, a self-processing tRNA-gRNA array was implemented (Xie et al., 2015). The tRNA-gRNA arrays were constructed using Gibson assembly. To generate this unique gRNA, overhang sequences were added to tracrRNA-tRNA templates from a previous study (Kwon et al., 2023; Invitrogen, Waltham, MA, USA) using primers 12/13 and 14/15 and PCR amplification to produce two gRNA fragments. The first gRNA fragment underwent an additional PCR step using primers 16/17 to add an additional tRNA overhang to allow for Gibson assembly. The gRNA fragments were added to the *BasI* site of a pENTR/D-TOPO (Thermo Fisher Scientific, Waltham, MA, USA) vector containing a synthetic gRNA expression cassette driven by the cauliflower mosaic virus 35S (35S) promoter using Gibson assembly. The completed gRNA expression cassette was then added to a binary destination vector containing a 35S driven Cas9 expression cassette using Gateway cloning, resulting in the CRISPR vector targeting *PsLOX2* used in this work.

### 5.3 Validation of gRNAs using mesophyll protoplasts

Tissue for preparation of mesophyll protoplasts was prepared from pea plants grown *in vitro.* First pea seeds were surface sterilized with 75% ethanol for 1 min in a 15 mL centrifuge tube. The ethanol was removed and then 10 mL of bleach solution was added. The seeds were incubated in the bleach solution for 15 min on a shaker. Seeds were then washed five time in sterilie water and placed on germination media (PhytoTech Labs; Product ID A 1375) in Magenta boxes and placed in a phytochamber. Leaf tissue from three week old plants was surface sterilized with 70% ethanol and cut into 0.5–1 mm strips using a fresh scalpel blade. The strips were then quickly transferred to a dish containing 10 mL of 0.6 M mannitol and incubated for 10 min on a shaker at 50 rpm in the dark. The mannitol solution was then removed and the leaf tissue was incubated in 10 mL of warm enzyme (37°C) solution consisting of 0.6 M mannitol, 10 mM MES, 1.5% Cellulase R-10, 0.75% Macerozyme R-10, 10 mM CaCl_2_, and 0.1% BSA. The dish was placed back onto the shaker at 50 rpm for 4 h in the dark. Protoplasts freed from the leaf tissue were isolated with a 40 μm filter, and washed with W5 consisting of 5 mM KCl, 154 mM NaCl, 125 mM CaCl_2_, and 2 mM MES pH 5.7, and added to the top of 5 mL of 0.55 M sucrose in a round bottom culture tube (Fisher Scientific, Hampton, NH). Protoplasts were further purified by centrifugation for 10 min at 100 g and 4°C in a swinging bucket rotor without using brakes. A green intermediate layer formed and was carefully transferred to a sterile round bottom culture tube. Then 7 mL of W5 solution was carefully added to the protoplasts followed by another centrifugation for 5 min at 100 g with brakes on. The supernatant was discarded and 7 mL of W5 solution was added to the purified protoplasts followed by a 2 min centrifugation at 100 g with brakes on. Lastly, the supernatant was removed and 1 mL of W5 solution was added to the purified protoplasts. A hemocytometer was used to estimate the number of protoplasts isolated.

MMG (0.5M mannitol, 15mM MgCl_2_ and 4mM MES; pH 5.7) solution was used to dilute the protoplasts to a concentration of 2 × 10^6^ protoplasts mL^−1^. Thirty µg of the *PsLOX2* CRISPR vector was added to a 2 mL microcentrifuge tube followed by 200 µL of the protoplast solution. The vector and protoplasts were then gently mixed. 230 µL of PEG solution was then added to the protoplasts and fully mixed with gentle tapping and inverting the tube several times. The mixture was then incubated for 30 min at room temperature in the dark. Two volumes (900 µL) of W5 solution was then added to the mixture and fully mixed by inverting the tube several times. This halted the transfection process. Finally, the transfected protoplasts were centrifuged for 5 min at 100 rpm.

Genomic DNA was extracted 72 h after transfection from the protoplasts and used to detect CRISPR-mediated mutations. *PsLOX2* was then amplified from the extracted protoplast genomic DNA as described above. The resulting PCR products were sequenced using primer 11. PCR products were determined to be mutated if their sequencing chromatograms contained mixed signals occurring near the double stranded break (DSB) site of either gRNA site.

### 5.4 Pea transformation and genotype analysis

Mutant *pslox2* pea lines were produced by *Agrobacterium*-mediated transformation of thin slices from developing embryo axes following Polowick et al. (2000) with slight modification. Colonies of *Agrobacterium tumefaciens* strain EHA105 harboring the CRISPR vector targeting *PsLOX2* were grown for 16–18 h at 28°C and 200 rpm in LB broth supplemented with 50 mg/L spectinomycin and 50 mg/L rifampicin. The overnight culture was pelleted by centrifugation at 3,000 rpm for 25 min. The pelleted cells were used to prepare infection media by resuspending them in MS media supplemented with 100 µM acetosyringone up to an OD_600_ of 0.2. Pea seeds cv. CDC Spectrum were surface sterilized, as described for protoplast isolation, and imbibed for 20 h in sterile water. Cotyledons were removed from the embryonic axes and explants were prepared by removing the top and bottom of the axes and slicing them four to five times perpendicular to their long axis. To prevent bacterial overgrowth, sterile filter paper was placed onto cocultivation media consiting of 1× B5 media, 3.0% sucrose, 0.8% phytoagar, 3 g/L KNO_3_, 770 mg/L CaCl_2_, 800 mg/L L-glutamine, 500 mg/L MgSO_4_·7H_2_O, 100 mg/L L-serine, 10 mg/L glutathione, 1 mg/L adenine, 1 mg/L 2,4-D, 0.2 mg/L kinetin, and pH 5.7. The explants were placed onto the filter paper and then covered with 5 µL of infection media and incubated for 4 days. Following co-cultivation, the explants were transferred to callus-inducing media consisting of 1× MS macro- and micronutrients, 1× B5 vitamins, 3.0% sucrose, 0.8% phytoagar, 2 mg/L BAP, 2 mg/L NAA, 150 mg/L Timentin^©^, 40 mg/L kanamycin, and pH 5.7. The explants were incubated for 2 weeks on callus-inducing media, after which the calli that formed were transferred to shoot-inducing media consisting of 1× MS macro- and micro-nutrients, 1× B5 vitamins, 3.0% sucrose, 0.8% phytoagar, 4.5 mg/L BAP, 0.02 mg/L NAA, 150 mg/L Timentin^©^, 50 mg/L kanamycin, and pH 5.7. Incubation for 6–9 weeks was required to induce shoot growth, with fresh media being supplied every 2 weeks. Shoots that were 0.5–1 cm in length were transferred to root-inducing media consisting of 0.5× MS, 3.0% sucrose, 0.8% phytoagar, 0.185 mg/L NAA, 150 mg/L Timentin^©^, and pH 5.7. When the shoots were 5–6 cm in length, with prolific root growth, the plantlets were transferred to soil. The plantlets in soil were transferred to resealable plastic bags to allow them to slowly harden by gradually opening the plastic bags over 2 weeks.

Genomic DNA was extracted from 20–50 mg of leaf tissue of plants brought out of tissue culture and T_2_ plants derived from them using the DNeasy Plant Mini Kit (Qiagen, Hilden, Germany). Plants were determined to be transgenic using primers 18/19 which are specific to the *neomycin phosphotransferase II* of the CRISPR vector. The *PsLOX2* target locus of transgenic plants was analyzed for mutations in the same way described for protoplasts.

### 5.5 Lipoxygenase assay

LOX assays were conducted in triplicates by preparing enzyme extracts from 100 mg of ground seeds (WT, TGP-2 to -9) with 1 mL of 50 mM sodium phosphate buffer pH 6.8 containing 4 mM sodium sulfite and 2 mM sodium ascorbate at 4°C for 4 h. Samples were centrifuged and subjected to fractionation to obtain a crude LOX fraction as described previously (Domoney et al., 1990). In brief, samples were brought to 25% saturation with ammonium sulfate, incubated on ice for 10 minutes, and centrifuged. Recovered supernatants were then brought to 60% saturation with ammonium sulfate and again incubated and centrifuged. The resulting pellets were resuspended in 500 µL of 50 mM sodium phosphate pH 6.8 giving the crude LOX fraction from the samples. This preparation was subjected to protein quantification by direct measurement of absorbance at 280 nm and used in the LOX assays described below.

LOX substrate was prepared by dissolving 4.5 µL linoleic acid (30 mM final concentration) in 500 µL of 50 mM sodium phosphate pH 6.8 containing 2% v/v Tween-20. Reactions were performed in triplicate and assembled by combining 200 µL of aerated 50 mM sodium phosphate pH 6.8, 5 µL of the crude LOX fraction, and 2 µL of 30 mM linoleic acid substrate solution. Reactions were vortexed and the absorbance at 234 nm was measured immediately and again after five minutes on a Nanodrop One UV-Visual spectrophotometer. LOX activity was determined by subtraction of the initial from the final absorbance values divided by the exact time between measurements, with one unit of activity defined as an increase of 0.001 in absorbance at 234 nm with a path length of 1 cm. Data were combined and reported as the average with the standard error. Specific activity was calculated as unit activity per milligram protein (U/mg) in the partially purified LOX fraction with appropriate error propagation.

### 5.6 Fatty acid extraction and analysis by GC-FID

Lipids were extracted from pea flour samples (WT, TGP-2 to -9) based on Folch et al. (1957) with some modifications (Shariati-Ievari et al., 2016). The extracted methylated fatty acids were analyzed using a gas chromatograph coupled with a flame ionization detector (GC-FID). The GC-FID column was held at 100°C for 2 min before increasing to 175°C at 25°C/min for 30 min. The column temperature was then increased to 220˚C at 15°C/min and held for another 10 min. The final temperature reached 240°C at 20°C/min and was held for 11 min. Each sample was run for a total of 60 min with a split ratio of 10:1 and a flow rate of 1.8 mL/min. Hydrogen was used as a carrier gas. A set of high-purity fatty acid standards (Nu-check; Funakoshi Co. Ltd.) was used for the quantification of fatty acids in each sample (Shariati-Ievari et al., 2016). Lipid extractions and fatty acid analysis were conducted in four replicates for each flour sample.

### 5.7 Extraction of volatile organic compounds (VOCs) using SPME fibers

Volatile extractions were performed using solid-phase microextraction (SPME) fibers (75µm Carboxen. PDMS, Supleco), with 5 g of each of the pea flour samples (WT, TGP-2 to -9) that were mixed with 10 g of NaCl and 90 mL of Milli-Q water in a 250 mL PYREX™ bottle. An internal standard, 1,2-dichlorobenzene (50 µL of 1 ng/mL in methanol), was added to the mixture before the SPME extraction. The Pyrex bottle was placed in a water bath held at 90°C (using CORNING PC-420D heater/magnetic stirrer) and the mixture was stirred carefully with a magnetic stirrer to prevent clump formation. A hole was made in the lid through which the SPME needle was introduced to the headspace above the mixture. The extraction was performed for 60 min.

### 5.8 GC-MS analysis of VOCs

Collected volatiles on SPME fibers were immediately analyzed using a 7890B GC with a 7693 Auto-Sampler connected to a 7000 GC/Triple Quadrupole mass spectrometry (MS) detector (Agilent Ltd, USA). The column was a HP-5MS (5%-phenyl)-methylpolysiloxane (Agilent Technologies; Santa Clara, CA, USA; 30 m × 250 μm × 0.25 μm film thickness). The carrier gas was helium (99.999% purity, Air Liquide Canada Inc., Montreal, QC, Canada) maintained at a constant flow (1 mL/min) with nitrogen (99.999% purity, Air Liquide Canada Inc.) as the make-up gas. The run was performed with the following temperature gradient, the initial 40°C temperature was held for 1 min, followed by a temperature increase of 4°C/min up to 200°C, a hold for 1 min, and a second increase of 10°C/min to 270°C which was held for 1 min for a total run of 54 min. The injector, at a temperature of 250°C, was operated in splitless mode with an injection volume of 1 μL. The Agilent Ltd. Triple Quadrupole MS was operated in electron impact (EI) mode with an ionization energy of 70 eV. The GC-MS transfer line and ion source temperatures were set at 250°C and 230°C, respectively. All analyses were performed in full scan mode with a scanning range of 29 to 500 m/z. A 3.7 min solvent delay was used to eliminate possible interfering solvent peaks from the chromatogram.

The semi-quantification of each volatile was calculated from the ratio of the base ion peak area for each VOC to the internal standard’s (m/z 146) base ion peak. The identity of each peak was determined by matching their mass spectra with the mass spectra of authentic compounds analyzed and reported in the National Institute of Standards and Technology (NIST version 2.3, 2017) library. The relative linear retention indices (LRIs) of each of the compounds were also calculated using the retention time obtained for a series of n-alkanes (C8–C20, 40 μg/mL hexane) as described previously (Aliani and Farmer, 2005).

### 5.9 E-nose sensor analysis

The WT and transgenic yellow pea flours (2g) were placed in a 20 mL glass tube covered with GC-MS vial septa lids where a hose equipped with a needle was placed for VOCs and chemical collection using a Sensigent MSEM 160. The collection was done over 60 sec with a pre-collection and purging time of 60 and 60 sec, respectively. Air was used as a blank and all analyses were performed using 5 replicates.

### 5.10 Statistical analysis

The differences in LOX activity, FAs, and VOCs in the different pea flours were determined using a one-way analysis of variance (ANOVA) followed by Tukey’s multiple comparison test. The relationship between LOX activity (Y variable) and the other measured parameters (X variables including the concentration of FAs, E-nose sensor response, and selected VOCs) was calculated using partial least squares regressions (PLS-R) using mean values for all variables (XLSTAT, version 19.4; Addinsoft, Paris, France). To visualize linear correlations between LOX activity and the concentration of the different VOCs and FAs in different pea flours, a correlogram was generated using R statistical package (version 4.02). The collected data was transferred into CDAnalysis software (Sensigent, Version 11.2), and processed using the specific parameters as follows: sensors ΔR/R as the data source, the baseline correction using the ‘Adv min max’ algorithm followed by normalization ‘Norm1’ and generation of the principal component analysis (PCA). A stepwise multiple regression analysis was performed using SPSS with all detected FAs, VOCs, and sensor responses to determine variables that can be used to predict the transgenic effect of reduced LOX activity.

## Data availability statement

The raw data supporting the conclusions of this article will be made available by the authors, without undue reservation.

## Author contributions

PB conceptualized the study and all authors contributed to the writing and critical review of the manuscript. MA conceptualized the study, a co-applicant for the NRC funding, analytical and statistical analysis, interpretation, writing, and critical review of the manuscript. SS-I provided technical support for extraction and analysis of fatty acids and volatile compounds. BP performed eNose sampling and LOX assay and WY conducted the pea transformation and plant regeneration CH and D-KR designed and cloned the CRISPR/Cas9 vector. All authors contributed to the article and approved the submitted version.
